# Service demand for psychological interventions among Australian adults: a population perspective

**DOI:** 10.1186/s12913-021-06101-3

**Published:** 2021-01-28

**Authors:** Imogen S. Page, Claudia Sparti, Damian Santomauro, Meredith G. Harris

**Affiliations:** 1 School of Public Health, University of Queensland, Brisbane, Australia; 2 Policy and Epidemiology Group, Queensland Centre for Mental Health Research, Brisbane, Australia; 3 Institute for Health Metrics and Evaluation, University of Washington, Seattle, USA

**Keywords:** Psychological interventions, Mental disorders, Health services, Survey, Epidemiology, Perceived need

## Abstract

**Background:**

Psychological interventions (PIs) are good practice treatment for both subthreshold and diagnosed mental disorders. Australia has implemented major reforms to expand the provision of subsidised psychological services for individuals with a diagnosed mental disorder. But there are gaps in knowledge about demand for PIs (i.e., use of and perceived need for PIs) across the population. This study uses nationally representative survey data from the 2007 Australian National Survey of Mental Health and Wellbeing to analyse demand for PIs. It also provides a method for analysing survey data to estimate demand for PIs when new survey data becomes available, along with suggestions to inform future survey development.

**Methods:**

Nationally representative community survey respondents (*n* = 8841, 16–85 years) indicated their perceived need for nine types of help for mental health problems in the past 12 months, including three PIs (cognitive behavioural therapy, psychotherapy, and counselling), and whether these needs were unmet, partially met, or fully met. Types of help were grouped as: PIs only; PIs plus other; and other only. Chi-square analyses were used to examine the association between type of intervention, sociodemographic and clinical factors, and type of professional consulted; multinomial logistic regression models were used to examine predictors of type of intervention(s) received.

**Results:**

7.9% (95%CI: 7.2–8.6) received PIs. Receipt of PIs was positively associated with higher education and consulting a mental health specialist. Twice as many respondents received PIs plus medication as compared to PIs only (4.2% vs. 2.0%). Almost half (45.4, 95%CI 36.5–54.6) incurred out-of-pocket costs for treatment. The most common reason for partially met need for PIs was cost (24.8, 95%CI 17.2–34.3); for unmet need, it was preference for self-management (33.9, 95%CI 21.2–49.5). Perceived unmet need for PIs only (3.1, 95%CI 2.1–4.6) or PIs plus other interventions (5.2, 95%CI 3.9–6.9%) was lower than for other interventions only (22.8, 95%CI 18.7–27.6).

**Conclusions:**

Continued reforms in Australia means that on-going monitoring of demand for PIs, using nationally representative data, is required. This study provides a baseline for comparison of the long-term effects of these reforms; this comparison may be undertaken using data from the third iteration of Australia’s NSMHWB, due for completion in 2021–22.

## Background

Psychological interventions (PIs) are talking therapies [1] and include cognitive behavioural therapy (CBT), psychotherapy, and counselling. Practice guidelines promote PIs as first-line treatments for subthreshold and diagnosed mental disorders [2–5]. Understanding service demand (the aggregate of service utilisation and unmet need for services) [6] for PIs is necessary for effective service planning. Knowing the level of demand allows planners to ensure there are sufficient resources (both staffing and structural) available to meet consumer needs and deliver evidence based care.

Studies show that demand for PIs is responsive to the health system in which they are delivered. For example, in the United States from 1987 to 2007, receipt of psychotherapy in the general population was stable but the number of consultations per person declined, while the use of psychopharmacological treatments increased. This likely reflected the promotion of medications and financial disincentives to providing psychotherapy at this time [7, 8]. In the United Kingdom, from 1991 to 2009, lower socioeconomic status was associated with greater odds of receiving publicly-funded PIs, whilst higher status was associated with greater odds of receiving privately-funded PIs [9]. In Canada, unmet need for PIs in 2002 was higher than other types of treatment [10], possibly because primary-care physicians are the most common providers of care and are better able to meet need for medication than PIs.

In 1997, data from Australia’s first national survey of mental health revealed significant receipt of, and unmet demand for, PIs. Findings revealed that 6.1% of adults had received PIs in the past year; equating to 55.1% of those who received help for mental health problems [11]. Among those who received help, 12.5% reported an unmet need for PIs; this was higher among people who self-identified as having depression or anxiety (a possible indicator of greater severity) [12]. People with diagnosed mental disorders more commonly received PIs if they consulted a mental health specialist or other health professional, than if they consulted only a general practitioner (GP) [13]. Of untreated adults with diagnosed depression or anxiety who perceived a need for treatment, PIs were the most wanted type of help [14].

In the following decade, Australia implemented major reforms that expanded the provision of subsidised psychological services: the Access to Allied Psychological Services program (ATAPS) launched in 2001 [15], and the Better Access to Psychiatrists, Psychologists and General Practitioners through the Medicare Benefits Schedule (Better Access) initiative launched in 2006 [16]. Analyses of data from a second national survey in 2007 revealed that, among adults who received any treatment for mental health problems, almost one-third still had an unmet need (15.1%) or partially met need (14.1%) for PIs [17]. Of those with a past year affective and/or anxiety disorder who sought treatment, only 46% received CBT from a health professional [18]. Among those with depression and/or anxiety disorder who had a Kessler Psychological Distress Scale [19] score ≥ 20, service demand was higher for PIs than other types of interventions, but perceived need for medication was more likely to be fully met than perceived need for PIs [20]. Cost (16.7%) and a preference for self-management (11.6%) were the main reasons for partially or unmet need for PIs [20].

These reforms have been ongoing, and have substantially affected the availability and uptake of PIs in Australia. Utilisation data does not provide a comprehensive picture of service demand because it is not from the consumer perspective and does not capture perceived or unmet need. Data is needed that captures demand in the entire community, not just those who meet criteria for a mental disorder. In 2019 the Australian Government committed to a new Intergenerational Health and Mental Health Study to begin in 2020 [21]. Data from the survey may provide insight into longer-term impacts of mental health reforms in Australia when compared against a relevant baseline. This paper provides a method for understanding the consumer perspective related to PIs and informs baseline findings to which this new survey data can be compared once available. Importantly, it also may be used to inform the design of the upcoming survey.

## Method

### Survey and sample

The second National Survey of Mental Health and Wellbeing (NSMHWB) was conducted by the Australian Bureau of Statistics in 2007 [22]. Respondents (16–85 years) were randomly recruited from a stratified, multistage probability sample of private dwellings. Face-to-face interviews were conducted; participants provided informed consent. In total, 8841 people were interviewed; the response rate was 60%. Due to the lower than expected response rate, possible bias in sample response was investigated by the ABS. The impact of non-response was found to be small at the aggregate level. Further detail on the non-response follow up study is provided in technical documentation [22]. The University of Queensland’s School of Public Health Research Ethics Committee approved the current study (approval number: IP16052016).

### Measures

#### Receipt of and perceived need for help

The survey instrument included the Perceived Needs for Care Questionnaire [23] which asked about need for different types of help for mental health problems over the past year, including PIs (i.e., CBT, psychotherapy, and counselling) and ‘other’ interventions (i.e., information, medications, and assistance with life skills with regards to money, work, looking after yourself, and meeting other people). A mutually exclusive, hierarchical ‘type of intervention’ classification was derived: PIs only; PIs plus other interventions; and other interventions only. Where respondents reported use of each type of help they were asked further questions on whether enough help was received (their perceived need for help). Categories of perceived need were ‘met’ or ‘partially met.’ Those whose needs were only ‘partially met’ indicated the main reason they did not get enough help. These reasons were grouped for analysis as either ‘structural’ (social, environmental or economic systems) or ‘attitudinal/knowledge’ (thoughts, feelings or ideas) barriers [24]. Respondents with a mental disorder diagnosis who did not receive help indicated whether they needed each type of help and to select (from the same list) the reasons they did not get that type of help (“Why didn’t you get this help?”).

Respondents indicated if they had consulted any of eight professionals for their health. These were recoded into four binary (yes/no) ‘type of professional’ variables: GP; psychologist; other mental health professional (i.e., psychiatrists, mental health nurses, and other professionals providing specialist mental health services); and other health professional (i.e., specialist doctors or surgeons, complementary/alternative therapists, and other professionals providing general services). If ‘yes,’ respondents indicated how many consultations with each professional were for mental health. Responses were collapsed into four groups (i.e., one, two to four, five to nine, and 10 or more) to ensure large enough cell counts to undertake statistical analyses. Respondents were also asked if they incurred out-of-pocket costs for each type of professional seen; a binary (yes/no) variable was derived.

#### Clinical characteristics

The World Mental Health Composite International Diagnostic Interview Third Edition (WMH-CIDI-3.0), was used to assess the presence of 12-month mental disorders according to the International Classification of Diseases, Tenth Revision (ICD-10) [25]: anxiety disorders (agoraphobia, social phobia, panic disorder, generalized anxiety disorder, obsessive compulsive disorder, and post-traumatic stress disorder); affective disorders (depression, dysthymia, and bipolar disorder), and; substance use disorders (harmful use and dependence derived separately for alcohol, cannabis, sedatives, stimulants and opioids).

Mental disorder severity (none, mild, moderate, and severe) was determined using an algorithm that calculated the impact of the disorder on functioning (accounting for comorbidity) [22]. The presence of any chronic physical condition in the past 12 months was categorised as a binary (yes/no) variable.

#### Disability

The World Health Organisation Disability Assessment Schedule (WHODAS) [26] assessed difficulties with performing tasks due to poor health over 30 days prior to the interview (0 = no disability to 100 = full disability). Respondents also indicated how many of the past 30 days they were unable to work or complete usual activities due to poor health.

#### Socio-demographic characteristics

Respondents’ age, sex, highest level of education, labour force status, marital status, geographical location (i.e., urbanicity), and Index of Relative Socioeconomic Disadvantage (IRSD) was recorded. Household financial problems in the past 12 months (i.e., could not pay electricity, gas or telephone bills on time; could not pay car registration or insurance on time; pawned or sold something; went without meals; unable to heat home; sought assistance from welfare/community organisations; or sought financial help from friends or family) was also recorded; a binary (yes/no) measure was derived.

### Data analysis

Data from the 2007 NSMHWB Basic Confidentialised Unit Record File (2009) (cat. no. 4326.0.30.002) were analysed using Stata MP version 13, accounting for the complex survey design and weighting procedures [27]. The jackknife method was employed to compute standard errors. Estimates with a relative standard error (RSE) of 0.25–0.50 were to be interpreted with caution; estimates with an RSE of > 0.50 were not reported [28]. A *p*-value <.05, and non-overlapping 95% confidence intervals, indicated statistical significance [28].

Weighted percentages and confidence intervals described help received and perceived need for help. Chi-square analyses examined the association between type of intervention, sociodemographic and clinical factors, and type of professional consulted. Multinomial logistic regression models examined predictors of type of intervention(s) received. The dependent variable was the hierarchical ‘type of intervention;’ the independent variables were sociodemographic and clinical and treatment factors. Given the relationship between provider and intervention received [29], two models were run with and without the type of professional consulted variable (Model 1 and Model 2). Independent variables were from previous studies [11, 30–33] and were considered if associated with the dependent variable (Wald *p* < .200) in univariate analyses [34]. Where correlations between independent variables were ≥ 0.40, the variable with better model fit was retained. Better model fit was determined by examining the effect the presence and absence of each variable, in each highly correlated pairs of variables, had on the overall model. Independent variables were also checked for outliers. Any cases with obvious outliers were removed from the analysis.

## Results

### Receipt of psychological interventions

In 2007, 7.9% of Australians received PIs in the past year, representing around two-thirds of those who received help for mental health problems. Of those receiving PIs, almost twice as many received counselling compared to CBT or psychotherapy. Receipt of PIs plus other interventions was more common than receipt of PIs only or other interventions only (see Table 1). Within the PIs plus other interventions group, the most common ‘other’ interventions received were medication (72.8, 95% CI: 65.5–79.2) and information (68.1, 95% CI: 62.9–73.0), followed by skills training (32.0, 95% CI: 26.3–38.3) and social interventions (22.2, 95% CI: 17.2–28.1); findings were similar in the other interventions only group (i.e., medication (86.1, 95% CI: 81.1–89.9); information (35.9, 95% CI: 29.0–43.5), skills training (7.8, 95% CI: 3.2–13.6); social interventions (5.4, 95% CI: 3.2–8.9)).

**Table 1:** Proportion of adults receiving help for their mental health in the past 12 months, and the types of help received (*N* = 8841)

Type of help received	% (95% CI)	n
**Psychological interventions**		
Psychotherapy	3.0 (2.5–3.6)	271
Cognitive Behavioural Therapy	3.4 (2.9–4.1)	326
Counselling	6.7 (6.1–7.3)	648
Any psychological intervention	7.9 (7.2–8.6)	759
**Information**		
Information about mental illness, its treatment, and available services	5.2 (4.6–5.9)	487
**Medication**		
Medicine or tablets	7.2 (6.6–8.0)	697
**Social interventions**		
Help to sort out housing or money problems	0.8 (0.5–1.1)	67
Help to meet people for support or company	0.9 (0.6–1.2)	80
Any social intervention	1.5 (1.1–1.9)	127
**Skills training**		
Help to improve your ability to work, or use your time in other ways	1.7 (1.3–2.2)	133
Help to improve your ability to look after yourself or your home	1.0 (0.7–1.3)	93
Any skills training	2.1 (1.7–2.6)	172
**Any help**	11.3 (10.5–12.3)	1101
**Intervention classification**		
Psychological interventions only	2.0^a^ (1.7–2.5)^c^	212
Psychological plus other interventions	5.8^a^ (5.2–6.5)^b^	547
Other interventions only	3.5 (3.1–3.9)^b^	342

*N* Unweighted number, denominator for this analysis; *n* Unweighted count, numerator; *%* Weighted percentage; *CI* Confidence interval

^a^ The sum of these groups is lower than the combined estimate of 7.9% in the upper section of Table 1, due to rounding

^b^ Non-overlapping confidence intervals indicate statistically significant differences between estimates

### Consultation characteristics

#### Main type of professional consulted

Psychologists (32.2, 95% CI: 27.6–37.2) and other mental health professionals (31.9, 95% CI: 27.6–36.6) were the main types of professional providing PIs (Fig. 1.1); GPs and other health professionals less commonly provided PIs than expected by chance. PIs plus other interventions were more commonly provided by GPs and other mental health professionals, and less commonly by psychologists, than expected by chance (χ^2^(3) = 31.76, *p* < .001, φ = .010).

**Fig. 1:**
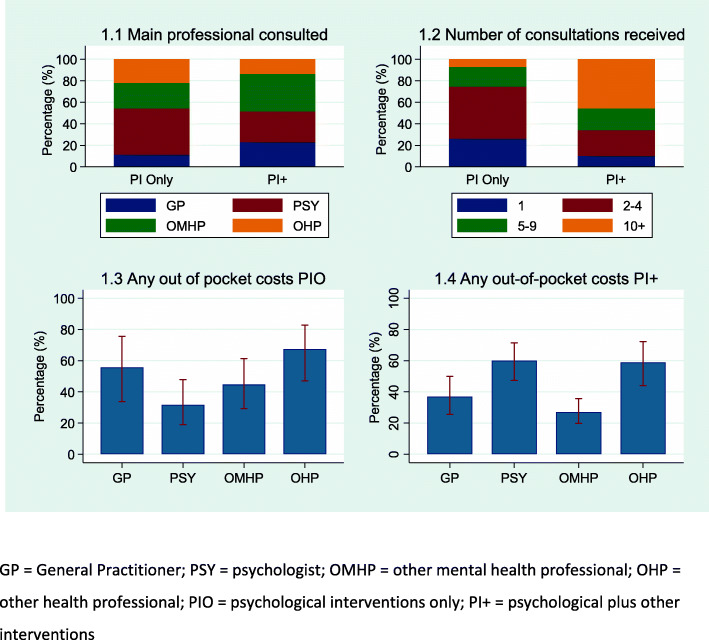
Consultation characteristics associated with receiving psychological interventions

#### Consultations received

Among respondents who received PIs only, around half (48.3, 95% CI: 37.9–58.8) received two to four consultations. Among those who received PIs plus other interventions, around half (45.7, 95% CI: 38.6–53.0) received ten or more (Fig. 1.2). The receipt of one, or two to four consultations, was more frequent in the PIs only group, and the receipt of ten or more consultations was less frequent in the PIs only group, than expected by chance (χ^2^(3) = 99.3, *p* < .001, φ = .009).

#### Out-of-pocket costs

Among respondents who received PIs only, or PIs plus other interventions, just under half incurred out of pocket costs (PIs only 45.4, 95% CI: 36.5–54.6; PIs plus other interventions 42.5 95% CI: 36.7–48.54). Incurring out-of-pocket costs varied depending on the main provider seen; however, differences were not significant (χ^2^(3) = 6.8, *p* = .078, φ = .002) (Figs. 1.3 and Fig. 1.4).

### Factors associated with the use of PIs

The multinomial model excluding type of professional consulted (Model 1 Table 2) showed two to three times higher odds of receiving PIs only among those with more education. Being in the second-most disadvantaged quintile of the IRSD (compared to the most disadvantaged), and having a 12-month affective disorder or a 12-month anxiety disorder doubled the odds of receiving PIs plus other interventions. In the model including type of professional consulted (Model 2, Table 2) consulting a mental health professional or other health professional increased the odds of receiving PIs only and PIs plus other interventions. Relative socio-economic disadvantage was no longer a significant predictor of receiving PIs plus other interventions.

**Table 2:** Socio-demographic, clinical and treatment factors associated with type(s) of intervention received among adults population who received any help for mental health problems over the past 12 months (*N* = 1101)

Predictor variable^**b**^	Model 1^**a**^		Model 2^**a**^	
	PIo	PI+	PIo	PI+
Age				
16–24	1.2 (0.5–3.3)	1.0 (0.4–2.3)	1.3 (0.5–3.6)	1.1 (0.5–2.5)
25–34	0.9 (0.4–2.2)	1.9 (0.9–3.9)	1.1 (0.5–2.8)	2.3 (1.2–4.6)
35–44	1.0	1.0	1.0	1.0
45–54	0.9 (0.4–2.4)	1.6 (0.8–3.2)	0.8 (0.3–2.1)	1.6 (0.7–3.3)
55+	0.7 (0.3–1.7)	1.2 (0.6–2.4)	0.9 (0.3–2.5)	1.9 (0.8–4.4)
Highest educational attainment				
Completed grade 10 or below	1.0	1.0	1.0	1.0
Completed grade 11 or 12	2.5 (1.2–5.0)*	1.4 (0.7–3.1)	2.3 (1.1–5.1)*	1.5 (0.6–3.8)
Completed higher education (tertiary education)	3.1 (1.6–6.0)*	1.5 (0.9–2.6)	2.7 (1.3–5.5)*	1.4 (0.7–2.5)
Labour force status				
Employed	1.0	1.0	1.0	1.0
Unemployed	0.7 (0.3–1.6)	0.9 (0.5–1.5)	0.6 (0.2–1.4)	0.6 (0.3–1.3)
Marital status				
Never married	1.0	1.0	1.0	1.0
Married	1.1 (0.4–3.1)	0.7 (0.3–1.5)	1.1 (0.4–3.2)	0.7 (0.3–1.4)
Separated/widowed/divorced	0.8 (0.3–2.0)	0.7 (0.3–1.6)	0.9 (0.3–2.6)	0.8 (0.3–1.9)
Index of disadvantage (quintiles)				
1 (most disadvantaged)	1.0	1.0	1.0	1.0
2	0.8 (0.3–2.0)	2.1 (1.1–4.0)*	0.7 (0.3–1.9)	1.8 (0.9–3.6)
3	0.8 (0.3–1.7)	1.3 (0.7–2.7)	0.6 (0.2–1.7)	1.1 (0.5–2.6)
4	1.3 (0.5–3.7)	1.8 (0.8–3.7)	1.0 (0.4–2.8)	1.4 (0.6–3.2)
5 (least disadvantaged)	0.9 (0.4–2.4)	1.7 (0.9–3.3)	0.7 (0.2–2.1)	1.3 (0.7–2.7)
Financial problems over past 12 months				
No	1.0	1.0	1.0	1.0
Yes	0.8 (0.4–1.8)	1.3 (0.8–2.0)	0.8 (0.3–1.9)	1.3 (0.8–2.2)
12-month affective disorder				
No	1.0	1.0	1.0	1.0
Yes	0.8 (0.5–1.4)	2.0 (1.2–3.1)*	0.9 (0.5–1.6)	2.0 (1.2–3.5)*
12-month anxiety disorder				
No	1.0	1.0	1.0	1.0
Yes	1.5 (0.9–2.4)	1.5 (1.0–2.4)*	1.2 (0.7–2.3)	1.2 (0.7–2.1)*
12-month substance use disorder				
No	1.0	1.0	1.0	1.0
Yes	1.7 (0.5–6.0)	1.4 (0.6–3.5)	1.5 (0.4–6.3)	1.2 (0.3–4.6)
WHODAS score	0.98 (0.95–1.00)	0.98 (0.97–0.99)	0.97 (0.96–0.99)	0.98 (0.96–0.99)
Professionals consulted for mental health in past year				
GP only	–	–	1.0	1.0
Mental health specialists	–	–	25.2 (11.7–54.4)*	21.0 (11.6–38.2)*
Other health professionals			12.7 (4.4–37.1)*	5.8 (2.2–15.2)*

Model 1: ‘professionals consulted’ variable not included; Model 2: ‘professionals consulted’ variable included

*PIo* Psychological interventions only; *PI+* Psychological plus other interventions

*N* Unweighted number, denominator for this analysis

^a^ Reference category: Other interventions only (OIo)

^b^ Sex, urbanicity, 12-month physical condition, and ICD disorder severity did not meet the threshold for inclusion in the model or were excluded due to issues of collinearity

### Perceived need and barriers to receiving PIs

Around one-quarter of those who received PIs reported partially met needs for PIs; the main reasons for not receiving further PIs were “I couldn’t afford it” and “I preferred to manage myself” (Table 3).

**Table 3:** Perceived need for, and barriers to, psychological interventions

	% (95% CI)	n	% (95% CI)	n
	**Group A: Adults who received PIs in the past 12 months (*n*** **= 759)**		**Group B: Adults with a likely mental disorder diagnosis who did not receive any type of help for mental health in the past 12 months (*n*** **= 1120)**	
**Whether needs for PIs were met, by interventions received**				
Received PIo – partially met need	23.1 (15.6–32.8)	47	–	–
Received PI+ − partially met need	19.6 (15.2–24.8)	112	–	–
Received PIo – met need	76.9 (67.2–84.4)	165	–	–
Received PI+ − met need	80.4 (75.2–84.8)	435	–	–
**Whether perceived a need for specific types of help**				
PIo	–	–	3.1 (2.1–4.6)	40
PI+	–	–	5.2 (3.9–6.9)	58
Other interventions	–	–	22.8 (18.7–27.6)	244
No need	–	–	68.9 (64.1–73.4)	778
	**Main reason for not receiving more PIs among the subset of Group A who reported partially met need (*n*** **= 157)**^**a**^		**Main reason for not receiving PIs among the subset of Group B with perceived need for PIs (*n*** **= 96)**^**a**^	
**Structural barriers**				
“I couldn’t afford the money”	24.8 (17.2–34.3)	45	9.5^b^ (4.8–18.1)	12
“I asked but didn’t get help”	19.4^b^ (9.5–35.4)	24	25.7 (15.6–39.2)	21
“I got help from another source”	3.7^b^ (1.6–7.8)	7	- ^c^	9
*Any structural barrier*	47.7 (36.4–59.2)	76	43.8 (31.5–56.8)	42
**Attitudinal or knowledge barriers**				
“I preferred to manage myself”	19.9 (13.9–27.5)	35	33.9 (21.2–49.5)	30
“I didn’t think anything could help”	15.8^b^ (9.0–26.2)	24	10.2 (4.8–20.4)^b^	9
“I didn’t know where to get help”	7.2^b^ (2.7–17.8)	9	- ^c^	4
“I was afraid to ask for help, or what others would think of me if I did”	9.5^b^ (3.9–21.4)	13	9.2 (5.1–16.0) ^b^	11
*Any attitudinal or knowledge barrier*	52.3 (40.8–63.6)	81	56.2 (43.2–68.5)	54

*PIs* Psychological interventions; *PIo* Psychological interventions only; *PI+* Psychological plus other interventions; *N* Unweighted number, denominator for this analysis; *n* Unweighted number, numerator; *%* Weighted percentage; *CI* Confidence interval

^a^ Two respondents were excluded from analysis as their main barrier to receiving psychological interventions was unknown

^b^ Interpret with caution as this estimate has a relative standard error of 0.25–0.50

^c^ Estimate not reported because relative standard error was greater than 0.50

Around a third of respondents with a mental disorder, who did not receive help, perceived a need for help. Perceived needs for PIs were less common than for other interventions only. The main reasons for not receiving PIs were “I preferred to manage myself” and “I asked but didn’t get help” (Table 3).

## Discussion

These analyses of national survey data provide a baseline measure of demand for PIs taken early in the course of major national mental health reforms deigned to improve availability and access of PIs. They show that PIs are a therapeutic option used by 7.9% of Australian adults in the previous 12-months, representing around two-thirds of all adults who received help for mental health problems. Most received PIs plus other interventions, commonly medications. Those receiving PIs only had fewer consultations than those who received PIs plus other interventions; the probability of out-of-pocket costs was just under 50% for both groups. Use of PIs was lower among people with less education and those who consulted only a GP. Among those who received PIs, cost was the main barrier to receiving further PIs. Among those who did not receive help, but felt they needed PIs, a preference for self-management was the main barrier to receiving PIs.

### Limitations

There are limitations to the current study. First, the survey used a self-report measure of service use which may result in under-reporting [35]. However, one study found that estimates of service use from the NSMHWB corresponded with independent counts [36]. Second, treatment rates for mental disorders in Australia have increased since the 2007 NSMHWB - from 37 to 46% in 2009–2010, largely due to the reforms in psychological therapy provision [37]. Nonetheless, the survey was completed 5 years after the introduction of ATAPS, and 1 year after Better Access, therefore providing insight into the influence of these reforms. The future Intergenerational Health and Mental Health Study [21] may be able to provide insight into longer-term impacts of reforms. Third, the survey had a 60% response rate; nonetheless, this is within the range of comparable surveys [38]. Fourth, homeless and institutionalised people were excluded from the survey, meaning that demand for PIs may be underestimated.

### Implications

The percentage of Australian adults who received PIs was higher (7.9%) than other high-income countries at a similar time-point (2.1–3.9%) [8, 9, 39]. This may reflect the narrower scope of PIs captured in US [7, 8] and UK studies [9]. It might also reflect the increased availability of PIs in Australia under new reforms [40] and/or improved mental health literacy leading to greater help seeking in the community over the preceeding decade [40].

PIs were twice as likely to be received in combination with medications than alone (PIs only 2.0% vs. PI plus medications 4.2%). Practice guidelines [41] suggest that medications should be prescribed for moderate to severe mental disorder diagnoses and PIs for most if not all mental disorder diagnoses across all severity levels, and subthreshold disorders. Considering that around two times as many Australians have a subthreshold/mild disorder (22.3%) compared to a moderate/severe disorder (12.0%) [42]), we might expect more people to be receiving PIs only rather than PIs plus medications. Concerns have been raised about an increase in combined treatments given the lack of corresponding population-level health gains and it has been suggested that combined treatment will only be of benefit if there are improvements in the targeted prescription of medications and an increase in the quality of PIs delivered [43].

PIs were commonly received via a mental health specialist. However, a significant minority of those consulting only GPs received PIs. Given this pattern was present in 1997 [13], it needs to be ensured that those who consult only a GP–whether because of preference or access issues - receive PIs if indicated [18].

Around half of respondents who received PIs reported no out-of-pocket costs for their treatment. Given that, before the reforms, the cost of therapy from a psychologist was borne by the recipient, this may reflect the impact of funding reforms in reducing cost-related barriers to access. The 2007 NSMHWB captured out-of-pocket costs in dollars, but this data was based on provider consulted, rather than intervention received, and for individuals who had used PIs only cell counts were small and could not be used to generate reliable estimates. Ensuring the upcoming survey captures the costs assocated with intervention received, rather than only by provider type would offer further insights into how PIs are being used in Australia.

As found elsewhere [29, 44–46], people with higher education levels were more likely to receive PIs only (as compared to ‘other’ interventions only). This group may have a better understanding of the potential benefits of PIs, or may define their health more broadly [44] and/or view the use of said help more positively [45]. Having an affective or anxiety disorder increased the odds of receiving PIs plus other interventions. Given three-quarters of the latter group received medications this may reflect best practice guidelines recommendations for combination treatment for moderate/severe disorders [41]. When ‘type of professional consulted’ was included in the model, the effect of socio-economic disadvantage disappeared. This suggests that the distribution of different professionals across different socioeconomic strata is unequal. This interpretation is supported by data from the 2017–18 Australian National Health Survey, which shows the proportion of people reporting having no specialists nearby increases as remoteness increases [47].

As in 1997 [17], most respondents who received PIs reported this need to have been fully met, suggesting these interventions continue to be acceptable to patients. The current study extends previous work by showing that cost was a common barrier to receiving ‘enough’ PIs and that around half of people receiving PIs incurred out-of-pocket costs. Analysis of data on claims for Better Access psychological therapy services could shed light on this by examining whether out-of-pocket costs at initial consultation reduce the likelihood of further consultations. In contrast, a preference for self-management was the most common barrier to accessing PIs among those with unmet need and was also common among those with partially met needs. This may suggest a role for digital PI modalities in this group [48]. ‘I asked but didn’t get help’ was also a common barrier in both groups; further research is needed to unpack this finding.

## Conclusion

In Australia in 2007, PIs were a common form of mental health intervention. However, evidence suggests that, in the early stages of reform, some groups (people with less education, and those consulting only a GP) may have been missing out and that efforts to address cost barriers had not been fully realised. PIs were most commonly received in combination with medication; at a population level, this may indicate a mismatch between actual and recommended treatment. A similar analysis of data from the planned third national mental health survey may enable the full effects of these reforms to be examined. The field dates for the next survey will also mean the current study provides a baseline against which provision of PIs via telehealth during the COVID-19 pandemic has affected cost and other barriers to access and met need. For this reason, we suggest this future survey should capture consultation characteristics such as out-of-pocket costs according to intervention received (rather than by provider). This would allow for further monitoring of the combined use of PIs plus medications (or other combinations of PIs and other interventions as necessary). Further research is suggested to disentangle the role of payment method and out-of-pocket costs in shaping patterns of receipt of PIs.

## Data Availability

The datasets generated and/or analysed during the current study are available in the ABS repository. To access this data you need to be an approved researcher with a login. Details on the 2007 National Survey of Mental Health and Wellbeing are available here: https://www.abs.gov.au/ausstats/abs@.nsf/mf/4326.0.

## Abbreviations

ATAPS: Access to Allied Psychological Services Program
Better Access: Better Access to psychiatrists, psychologists and general practitioners through the medicare benefits schedule
CBT: Cognitive behavioural therapy
GP: General practitioner
ICD-10: International Classification of Diseases, tenth revision
IRSD: Index of relative socioeconomic disadvantage
NSMHWB: National Survey of Mental Health and Wellbeing
PIs: Psychological interventions
RSE: Relative standard error
WHODAS: World Health Organisation Disability Assessment Schedule
WMH-CIDI-3.0: The World Mental Health Composite International Diagnostic Interview third edition
